# Weakness acquired in the intensive care unit. Incidence, risk factors and their association with inspiratory weakness. Observational cohort study

**DOI:** 10.5935/0103-507X.20170063

**Published:** 2017

**Authors:** Ladislao Pablo Diaz Ballve, Nahuel Dargains, José García Urrutia Inchaustegui, Antonella Bratos, Maria de los Milagros Percaz, Cesar Bueno Ardariz, Sabrina Cagide, Carolina Balestrieri, Claudio Gamarra, Dario Paz, Eliana Rotela, Sebastian Muller, Fernando Bustos, Ricard Aranda Castro, Esteban Settembrino

**Affiliations:** 1 Hospital Nacional Profesor Alejandro Posadas - Buenos Aires, Argentina.; 2 Universidad Nacional de la Matanza - Buenos Aires, Argentina.; 3 Clínica Olivos - SMG - Olivos, Buenos Aires, Argentina.

**Keywords:** Muscle weakness, Respiration, artificial, Delirium, Maximal respiratory pressures, Hyperglycemia, Debilidad muscular, Respiración artificial, Delirio, Presiones inspiratorias máximas, Hiperglucemia

## Abstract

**Objective:**

This paper sought to determine the accumulated incidence and analyze the risk factors associated with the development of weakness acquired in the intensive care unit and its relationship to inspiratory weakness.

**Methods:**

We conducted a prospective cohort study at a single center, multipurpose medical-surgical intensive care unit. We included adult patients who required mechanical ventilation ≥ 24 hours between July 2014 and January 2016. No interventions were performed. Demographic data, clinical diagnoses, the factors related to the development of intensive care unit -acquired weakness, and maximal inspiratory pressure were recorded.

**Results:**

Of the 111 patients included, 66 developed intensive care unit -acquired weakness, with a cumulative incidence of 40.5% over 18 months. The group with intensive care unit-acquired weakness were older (55.9 ± 17.6 *versus* 45.8 ± 16.7), required more mechanical ventilation (7 [4 - 10] days *versus* 4 [2 - 7.3] days), and spent more time in the intensive care unit (15.5 [9.2 - 22.8] days *versus* 9 [6 - 14] days). More patients presented with delirium (68% *versus* 39%), hyperglycemia > 3 days (84% *versus* 59%), and positive balance > 3 days (73.3% *versus* 37%). All comparisons were significant at p < 0.05. A multiple logistic regression identified age, hyperglycemia ≥ 3 days, delirium, and mechanical ventilation > 5 days as independent predictors of intensive care unit-acquired weakness. Low maximal inspiratory pressure was associated with intensive care unit-acquired weakness (p < 0.001), and the maximum inspiratory pressure cut-off value of < 36cmH_2_O had sensitivity and specificity values of 31.8% and 95.5%, respectively, when classifying patients with intensive care unit-acquired weakness.

**Conclusion:**

The intensive care unit acquired weakness is a condition with a high incidence in our environment. The development of intensive care unit-acquired weakness was associated with age, delirium, hyperglycemia, and mechanical ventilation > 5 days. The maximum inspiratory pressure value of ≥ 36cmH_2_O was associated with a high diagnostic value to exclude the presence of intensive care unit -acquired weakness.

## Abstract (es)

**Objetivo:**

Conocer la incidencia acumulada y analizar los factores riesgo asociados al desarrollo de debilidad adquirida en la unidad de cuidados intensivos y su asociación con la debilidad inspiratoria.

**Métodos:**

Estudio de cohorte prospectivo en un solo centro, unidad de cuidados intensivos médico-quirúrgica polivalente. Se incluyeron pacientes adultos, que hayan requerido ventilación mecánica ≥ 24 horas entre julio de 2014 y enero de 2016. No hubo intervenciones. Se registraron datos demográficos, diagnóstico clínico y factores relacionados con el desarrollo de debilidad adquirida en la unidad de cuidados intensivos y Presión inspiratoria máxima.

**Resultados:**

Ciento once pacientes incluidos, 66 desarrollaron debilidad adquirida en la unidad de cuidados intensivos, con una incidencia acumulada del 40,5% en 18 meses. El grupo con debilidad adquirida en la unidad de cuidados intensivos presentó mayor edad (55,9 ± 17,6 *versus* 45.8 ± 16.7), además de más días con ventilación mecánica (7 [4 - 10] *versus* 4 [2 - 7,3]), más días en unidad de cuidados intensivos (15,5 [9,2 - 22,8] *versus* 9 [6 - 14]). Hubo más pacientes con delirio (68% *versus* 39%), con hiperglucemia > 3 días (84% *versus* 59%); y con balance positivo > 3 días (73,3% *versus* 37%). Todas las comparaciones fueron significativas con p < 0,05. La regresión logística múltiple identificó a la edad, la hiperglucemia ≥ 3 días, el delirio y la ventilación mecánica > 5 días como predictores independientes para debilidad adquirida en la unidad de cuidados intensivos. La presión inspiratoria máxima baja se asoció a debilidad adquirida en la unidad de cuidados intensivos (p < 0,001) y el punto de corte presión inspiratoria máxima < 36cmH_2_O obtuvo una sensibilidad y especificidad del 31,8% y 95,5% para clasificar al grupo con debilidad adquirida en la unidad de cuidados intensivos.

**Conclusión:**

La debilidad adquirida en la unidad de cuidados intensivos es una condición con un alta incidencia en nuestro medio. El desarrollo de debilidad adquirida en la unidad de cuidados intensivos se asoció a la edad, delirio, hiperglucemia y la ventilación mecánica > 5 días. La presión inspiratoria máxima ≥ 36cmH_2_O demostró un alto valor diagnóstico para descartar la presencia de debilidad adquirida en la unidad de cuidados intensivos.

## INTRODUCTION

Intensive care unit (ICU)-acquired weakness represents an important clinical problem, and it is increasingly common among patients admitted to the ICU.^(1)^ This condition is characterized by a decrease in muscular strength; is generally associated with atrophy; has an acute onset; and is diffuse, symmetrical, and generalized. It develops after the onset of a critical illness, with no other identifiable cause. Intensive care unit-acquired weakness usually manifests bilaterally in the limbs with hyporeflexia or arreflexia and the preservation of the cranial nerves.^(2-4)^

Other common findings include a reduced cross-sectional area of muscle, decreased muscle protein synthesis with increased proinflammatory cytokine production, proteolysis, and muscle catabolism. In addition, the deterioration of the microvascular function, which is associated with resistance to insulin, is usually described.^(5)^

Intensive care unit-acquired weakness and its associated neuromuscular dysfunctions are detected in 25-50% of patients who require more than 5 days of invasive mechanical ventilation (MV),^(6)^ which is associated with difficulty in weaning, a prolonged stay in the ICU, and increases in morbidity and mortality.^(7-9)^ In turn, it can persist for years after discharge and affect patient quality of life.^(10,11)^

The etiology of ICU-acquired weakness is multifactorial and related to various risk factors such as prolonged MV, ICU stay, prolonged immobility, the use of neuromuscular blockers or corticoid therapy, hyperglycemia, shock, sepsis, and renal failure.^(2,10,12,13)^

Intensive care unit-acquired weakness is not limited to the muscles of the extremities. Powers et al. observed that atrophy of the diaphragmatic musculature occurs 18 hours after the initiation of controlled MV and has been described as a cause of delayed ventilatory weaning; conversely, the same level of atrophy occurs in the skeletal muscles of the extremities after 96 hours of controlled MV.^(8)^

Currently, no consensus exists regarding the gold standard for the diagnosis of ICU-acquired weakness.^(14)^ Different methods are used to identify this clinical picture, including muscular biopsy, electromyogram, and the skeletal muscle strength assessment of the Medical Research Council (mss-MRC). Both muscular biopsies and electromyograms are invasive tests with limitations for application in the ICU and should be used to define or clarify a diagnostic suspicion; however, their usefulness as a research method is limited.^(12,14,15)^ The simplest and most widely accepted tool for diagnosing ICU-acquired weakness is the mss-MRC.^(12,16-18)^ The force of inspiratory muscles is measured via maximum inspiratory pressure (Pimax).^(19,20)^

The current study sought to calculate the cumulative 18-month incidence of ICU-acquired weakness among patients admitted to a medical/surgical ICU. In addition, we analyzed whether the variables identified as risk factors were associated, both jointly and independently, with the development of ICU-acquired weakness. Secondarily, we assessed the relationship between ICU-acquired weakness and inspiratory muscle weakness via Pimax.

## METHODS

A prospective cohort study was conducted at a single institution. The study protocol was presented to and approved by the Teaching and Research Committee and the "Dr. Vicente Federico del Giudice" Bioethics Committee of the *Hospital Nacional Profesor Alejandro Posadas*.

The study was performed at a multipurpose ICU with 26 beds. This unit receives patients with both medical and postoperative pathologies from a general acute care hospital. Patients > 18 years of age hospitalized in the ICU who required invasive MV for > 24 hours were included between July 2014 and January 2016. The patient or relative in charge provided informed consent to participate in this study. Patients with central or peripheral nervous system injury, motor sequelae as a reason for admission, histories of neuromuscular disease, antecedents of cognitive disorders that prevented the understanding of simple orders, orthopedic or traumatic limitations upon admission, or a Barthel score < 35 points the week prior to admission to the ICU (referred by the patient or family member) were excluded.

The variables measured in this study included age, gender, reason for ICU admission, previous history of ICU admission, and the Barthel index, which was completed by questioning the patients or their next of kin by asking about the week prior to ICU admission. The following factors related to the development of weakness were collected each day: days receiving analgesics, days under sedation, days with interrupted sedation, days with renal failure (plasma creatinine ≥ 1.2mg/dL and/or hemodialysis requirement), days receiving vasopressor drugs (continuous or intermittent administration), and treatment with antibiotics. The following dichotomous variables that were known cut-off points previously identified were collected: MV > 5 days, neuromuscular blockers (2 or more days of blockers), hyperglycemia (the presence of ≥ 3 consecutive days with a plasma glucose value ≥ 150mg/dL per glucose test that required correction with intravenous insulin), prolonged corticosteroid therapy (≥ 3 days using any corticoid), positive balance (≥ 3 consecutive days with total excretion less than ingestion), the positive presence of delirium ([Confusion Assessment Method of the Intensive Care Unit - CAM-ICU] at least once a day),^(11)^ Pimax (in cmH_2_O) and the lower limit of normality (minimum theoretical value of Pimax for each patient in cmH_2_O, calculated using the Evans formula).^(12)^

Prior to using the mss-MRC, the state of alertness was assessed using the Richmond Agitation-Sedation Scale (RASS), the values of which should range between 1 and -1. The infusion of sedatives was discontinued at least 30 minutes prior to applying the mss-MRC. The compression capacity was assessed by asking the patient to perform between 4 and 6 simple commands: "Open your eyes" or "Close your eyes" (as appropriate), "Lift your eyebrows", "Move your head to one side (or the other)", "Squeeze my hand", "Open your mouth", and "Stick out your tongue". After four of these commands were performed, muscle force was evaluated using the mss-MRC (Appendix 1).

**Figure 1:**
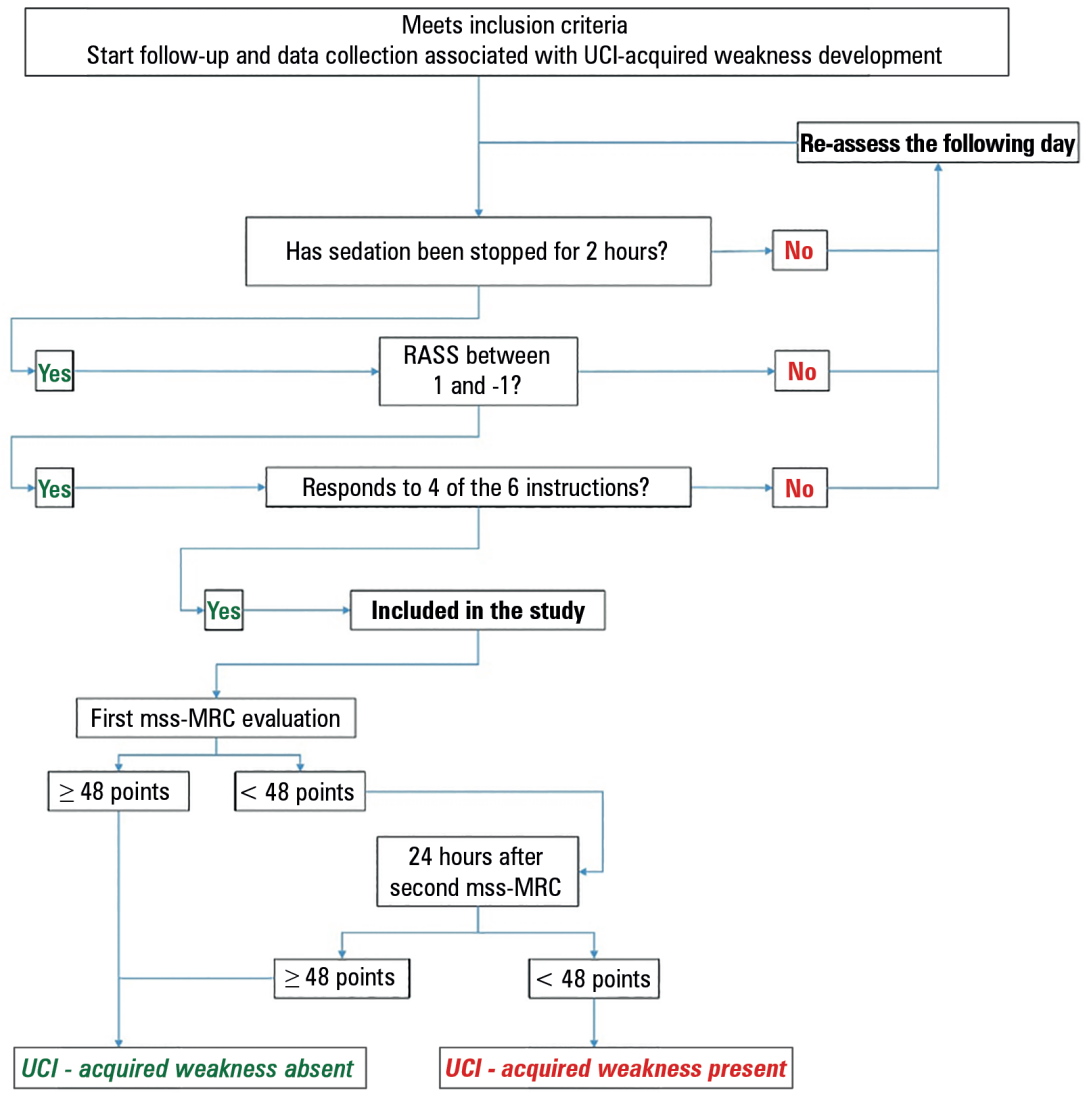
Flowchart of the procedures performed in this study. ICU - intensive care unit; mss-MRC - muscular strength scale of the Medical Research Council.

Figure 1 shows the method for arriving at a diagnosis. The patient was classified as "without ICU-acquired weakness" when he or she reached ≥ 48 points or was considered as "re-assessable" when the cut-off point was not reached (i.e., mss-MRC < 48). During the morning of the following day, those who were "re-assessable" were given a second mss-MRC, which was performed by a different operator (who did not know the result of the first measurement). If the patient exceeded the cut-off point, then they were considered "without ICU-acquired weakness"; if, however, the blind evaluator obtained a value of < 48 points a second time, then the patient was considered to have ICU-acquired weakness.

Thirty minutes after the first mss-MRC measurement, the Pimax was determined. Patients sat at 45º, and a unidirectional valve aneroid manovacuometer was used to measure pressure. A nozzle interface was used for those without an artificial airway in place, and a 15mm adapter was used for patients with an orotracheal or tracheostomy tube. We quantified the Pimax achieved in 20 seconds,^(21)^ and the highest value of three replicates was reported. The inter-observer reliability of different consecutive operators was measured using a subsample of the first 10 patients whose mss-MRC and Pimax assessments were repeated.

### Statistical analyses

The results of the categorical variables are presented as counts and proportions within their categories. The numerical variables, whether continuous or discrete, are presented according to their distribution as the means and standard deviations or medians and interquartile ranges.

The chi-square test or Fisher's exact test was used as appropriate to compare the association between categorical variables, and Student's t-test or Mann-Whitney U-test was used for numerical variables according to the distribution.

The inter-observer reliability for the performance of the mss-MRC in the diagnosis of ICU-acquired weakness (mss-MRC ≥ 48) was assessed using the agreement index for nominal variables (Cohen's Kappa), and the intra-class correlation coefficient (ICC) index was used for the Pimax.

To estimate the simultaneous effect of the variables identified as possible risk factors on the incidence of weakness, a conditional binary logistic regression model was used. Inclusion of the variables in the model was decided based on a p-value of < 0.1 in the univariate comparison. In addition, numerical variables that were significant in the univariate analysis and were previously individualized as clinically relevant subgroups were included dichotomously in the multivariate analysis (days of invasive MV > 5 days) for a better interpretation. A backward stepwise selection was used with Wald's method. The result of the multivariate binary logistic regression was expressed as an odds ratio (OR) with its corresponding 95% confidence intervals (95%CI).

The final calibration of the model was evaluated using the Hosmer-Lemeshow test, and the discriminating power was established based on an area under the curve (AUC) analysis.

A survival analysis using a Kaplan-Meier curve was used for the variables time to event (ICU-acquired weakness), and the subgroups with or without delirium as well as those with or without hyperglycemia were compared (i.e., the significant variables in the binary logistic regression analysis) relative to the development of ICU-acquired weakness over time. The log-rank test was used for comparisons among the subgroups.

The risk associated with a Pimax of 36cmH_2_O and its relationship to the clinical diagnosis of ICU-acquired weakness was calculated. In addition, the diagnostic performance of this cut-off point as well as the sensitivity, specificity, and positive and negative likelihood ratio (LR+ and LR-, respectively) of this parameter as a method to classify patients with ICU-acquired weakness was analyzed. The LR+ and LR- are reported because of their stability with respect to the possible variability in the prevalence of ICU-acquired weakness. Finally, the lower limit of normality was calculated to individualize the number of patients who did not reach the theoretical values for their age.

A value of p = 0.05 was considered significant. R version 3.1.3 was used to analyze the data.^(22)^

## RESULTS

**Figure 2:**
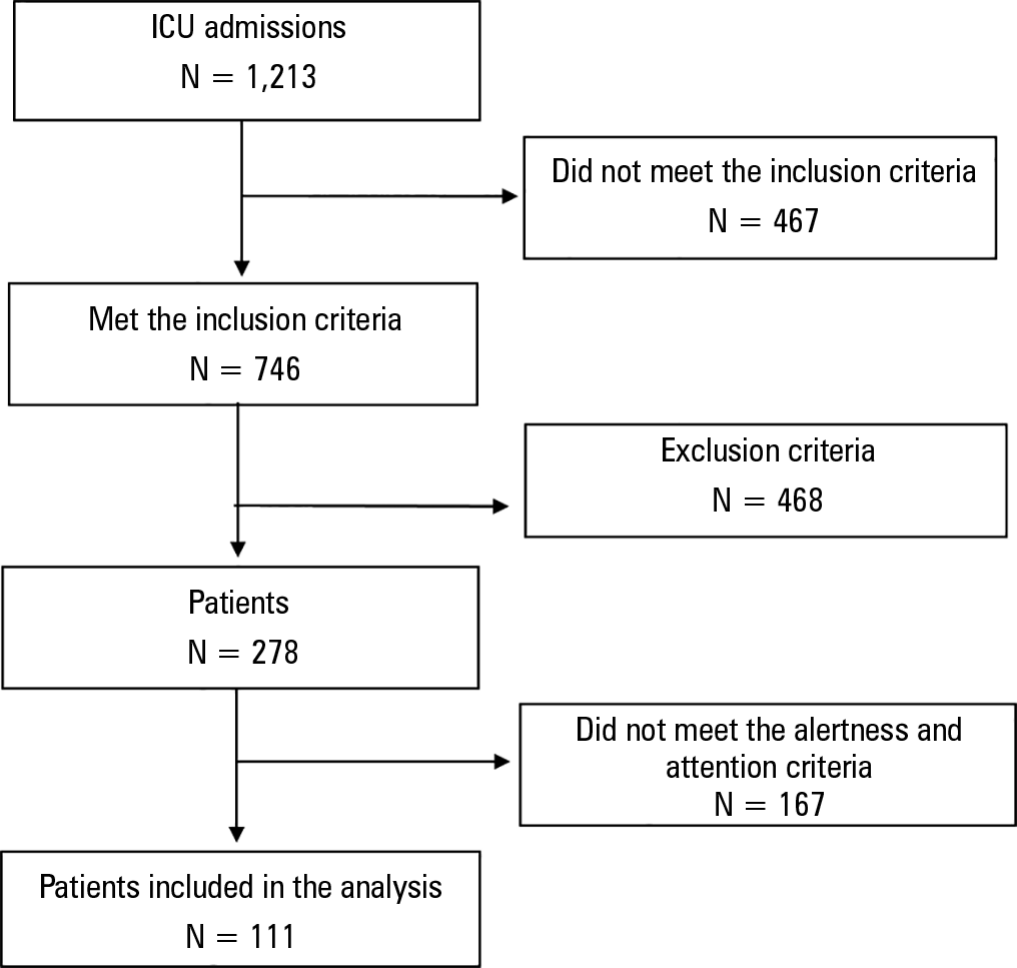
Flowchart of patients under study. ICU - intensive care unit.

A total of 111 consecutive patients were included (Figure 2), 66 of which were classified with "ICU-acquired weakness". A cumulative incidence of ICU-acquired weakness of 40.5% was observed after an 18-month follow-up period (95%CI = 31.8% - 49.8%). The incidence rate or density of ICU-acquired weakness was 0.0038 per patient per day of follow up. The maximum follow-up period for a patient was 156 days.

The characteristics of these patients are detailed in table 1. Significant differences (p < 0.05) were observed between patients with or without ICU-acquired weakness, as follows: age 55.9 (± 17.6) years *versus* 45.8 (± 16.7) years, respectively; median time with invasive MV 7 [4 - 10] days *versus* 4 [2 - 7.3] days, respectively; median time in the ICU 15.5 [9.2 - 22.8] days *versus* 9 [6 - 14] days, respectively; median time with sedation 2.5 [1 - 6] days *versus* 2 [0 - 3] days, respectively; median time with vasopressors 1 [0 - 3.75] day *versus* 1 [0 - 1.2] day, respectively; median time to renal failure 1 [0 - 23] days *versus* 0 [0 - 9.6] days, respectively; and median time receiving antibiotics 5.5 [3-9.75] days *versus* 4 [2.7 - 6] days, respectively. In addition, more patients had delirium (31 [68.9) *versus* 26 [39.4]], hyperglycemia > 3 days (37 [84.1) *versus* 39 [59.1]), corticosteroid therapy > 3 days (21 [46.7) *versus* 19 [18.8]], and positive balance > 3 days (33 [73.3) *versus* 25 [37.9]] in the ICU-acquired weakness group.

**Table 1:** Characteristics of the sample

Characteristics	ICU-acquired weakness		p value
	Yes N = 45	No N = 66	
Age†	55.9 ± 17.6	45.8 ± 16.7	0.004
Male	23 (51.1)	38 (57.6)	0.56
APACHE II	16.7 (5.1)	19.1 (7.3)	0.28
Barthel Score before ICU‡	100 [40 - 100]	100 [65 - 100]	0.82
Reasons for admission			
Doctor	31 (68.9)	46 (69.7)	0.99
Scheduled surgery	4 (8.9)	5 (7.6)	0.99
Emergency surgery	10 (22.2)	11(16.7)	0.47
Polytrauma/TEC	0 (0)	4 (6.1)	0.14
Main diagnoses			
Sepsis	11 (24.4)	21 (31.8)	0.52
Pneumonia	5 (11.1)	7 (10.6)	0.99
COPD	4 (8.9)	6 (9.1)	0.99
Asthmatic crisis	2 (4.4)	5 (7.6)	0.69
Abdominal surgery	11 (24.4)	9 (13.6)	0.20
Chest/cardiovascular surgery	1 (2.2)	6 (9.1)	0.23
Brain hemorrhage/neurosurgery	0 (0)	1 (1.5)	0.99
TBI	0 (0)	1 (1.5)	0.99
Diabetic ketoacidosis	3 (6.7)	2 (3.0)	0.39
Other	8 (17.8)	8 (12.1)	0.43
MV days	7 [4 - 10]	4 [2 - 7.3]	< 0.001
MV > 5 days	30 (66.6)	20 (30.3)	< 0.001
Reintubations	8 (17.7)	16 (24.2)	0.48
1 episode	5 (11.1)	7 (10.6)	
2 episodes	1 (2.2)	6 (9.1)	
3 episodes	2 (4.4)	3 (4.5)	
Days in ICU‡	15.5 [9.2 - 22.8]	9 [6 - 14]	< 0.001
Days with sedation‡	2.5 [1 - 6]	2 [0 - 3)	0.03
Days with analgesia‡	4 [2 - 8]	3 [1.7 - 6]	0.12
Days with window sedoanalgesia‡	2 [1 - 3]	2 [1 - 3)	0.31
Days with vasopressors‡	1 [0-3.75]	1 [0 - 1.2]	0.03
Days with renal failure‡	1 [0 - 23]	0 [0 - 9.6]	0.03
Days with antibiotics‡	5.5 [3 - 9.75]	4 [2.7 - 6]	0.049
Use of neuromuscular blockers	8 (17.8)	10 (15.2)	0.79
Hyperglycemia > 3 days	37 (84.1)	39 (59.1)	< 0.001
Corticotherapy > 3 days	21 (46.7)	19 (18.8)	0.07
Delirium (CAM-positive ICU)	31 (68.9)	26 (39.4)	0.004
Positive balance > 3 days	33 (73.3)	25 (37.9)	0.006
Pimax in cmH_2_O	41.6 ± 11.4	51 [50 - 51]	< 0.001
Pimax < 36cmH_2_O	15 (28.8)	3 (4.54)	< 0.001
Mortality in ICU	4 (8.8)	4 (6.1)	0.71

ICU - intensive care unit; APACHE II - Acute Physiology and Chronic Health Assessment II; TBI - traumatic brain injury; COPD - chronic obstructive pulmonary disease; MV - mechanical ventilation; CAM-ICU - Confusion Assessment Method for the Intensive Care Unit. Values are expressed in n (%) except where indicated.

† Mean ± SD;

‡ Median [Percentile 25-27].

The reliability between the five evaluators of the mss-MRC was measured using the data of the first 15 patients evaluated, and a Kappa value of 0.74 (95%CI = 0.51 - 0.97; p < 0.001) was obtained, showing "substantial"^(23)^ agreement to confirm or exclude ICU-acquired weakness. Likewise, the degree of agreement among the five evaluators for Pimax (in cmH_2_O) was measured, and an "excellent"^(24)^ agreement was obtained (ICC = 0.97; 95%CI = 0.93 - 0.99; p < 0.001).

Table 2 shows the results of the multivariate logistic regression analysis. The variables that were independently associated with the development of ICU-acquired weakness were age (OR = 1.03, 95%CI = 1.002 - 1.03, p = 0.035), hyperglycemia > 3 days (OR = 3.85, 95%CI = 1.28 - 11.54, p = 0.016), the presence of delirium (OR = 3.34, 95%CI = 1.31 - 8.50, p = 0.011), and invasive MV use > 5 days (OR = 2.83, 95%CI = 1.00 - 7.97, p = 0.049).

**Table 2:** Multivariate binomial logistic regression

Variables	OR	95%CI	p value
Age (years)	1.03	1.002 - 1.03	0.035
MV > 5 days	2.83	1.005 - 7.97	0.049
Delirium (CAM-positive ICU)	3.34	1.31 - 8.50	0.011
Hyperglycemia > 3 days	3.85	1.28 - 11.54	0.016

OR - odds ratio; 95%CI - 95% confidence intervals; MV - mechanical ventilation; CAM-ICU - Confusion Assessment Method for the intensive care unit.

The regression model showed a correct classification power of 73.6% regarding the events in the response variable.

The final logistic regression model obtained a correct calibration measured by the Hosmer-Lemeshow test (p = 0.854). Discrimination was classified as "good" assessed by the area under the ROC curve (AUC = 0.815, 95%CI = 0.73 - 0.89, p < 0.001).

**Figure 3:**
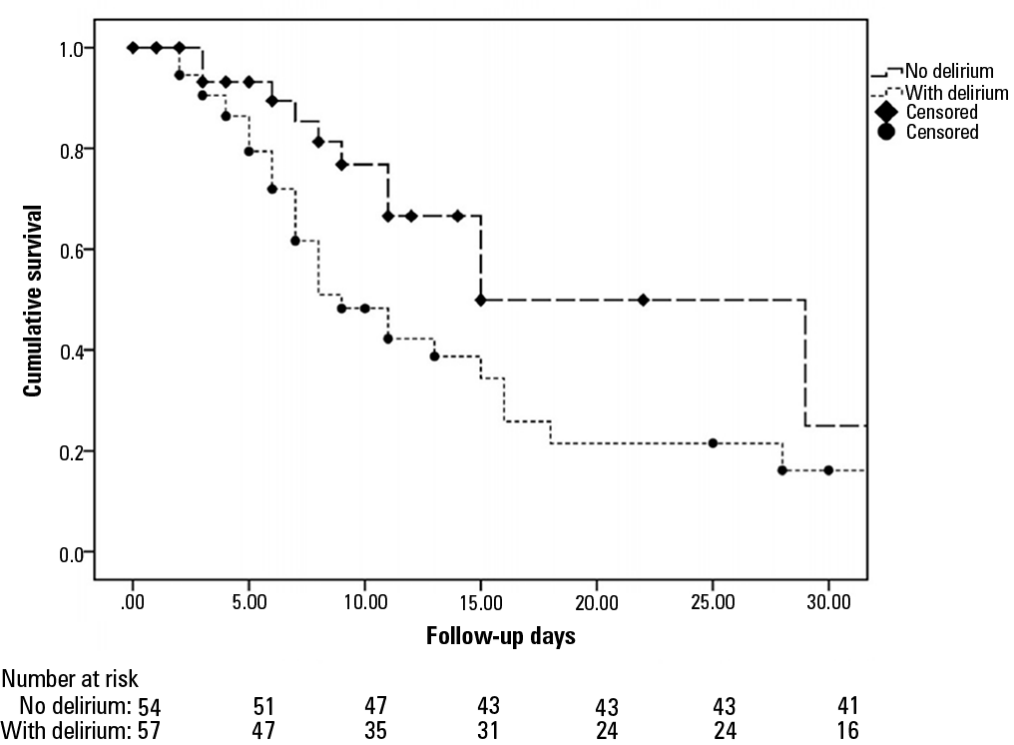
Kaplan-Meier curve. The likelihood of developing weakness in the presence of delirium (dotted line) *versus* no delirium (dashed line) after a 30-day follow-up period. (log-rank, p = 0.03).

The Kaplan-Meier curve (Figure 3) showed the probability of having ICU-acquired weakness depending on whether the patient had delirium during follow up. The groups that presented with delirium (dotted line) *versus* those that did not (dashed line) are shown. The comparison using the log-rank test was significant (p = 0.03). The probability of presenting with ICU-acquired weakness according to whether the patient had sustained hyperglycemia (> 3 days), a survival analysis, and a between-group comparison were not significant (log-rank test, p = 0.159).

**Figure 4:**
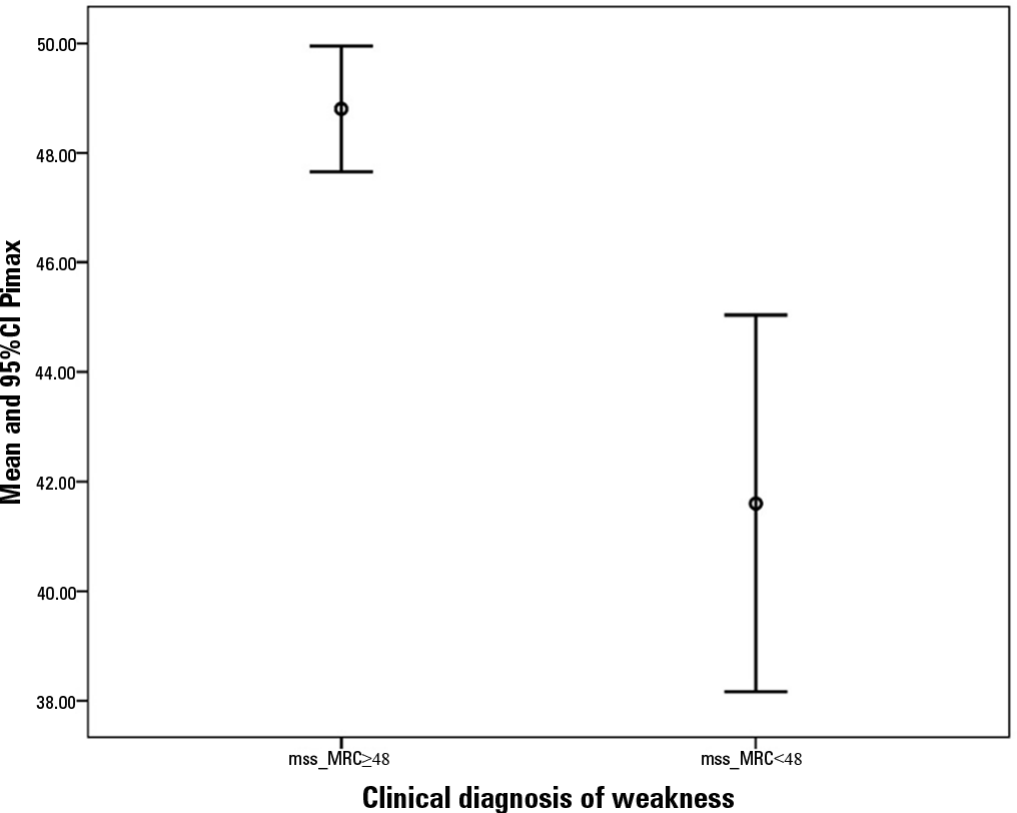
Error bars with means and 95% CIs of maximal inspiratory pressure regarding patients with Medical Research Council muscle strength scale values of ≥ 48 and < 48. 95%CI - 95% confidence intervals; Pimax - maximum inspiratory pressure; mss-MRC - muscular strength scale of the Medical Research Council.

Regarding inspiratory muscle strength, the absolute Pimax values were compared between the group that developed ICU weakness, 41.6 (± 11.4) cmH_2_O, and the group that did not, 48.8 (± 4.67) cmH_2_O (p < 0.0001; Figure 4). The cut-off value described above (Pimax < 36cmH_2_O *versus* Pimax ≥ 36 cmH_2_O) showed that 30 (66%) of the 45 patients who developed ICU-acquired weakness fell below the cut-off point; on the other hand, only 15 (22%) patients in the group that did not have a clinical diagnosis of ICU-acquired weakness obtained a Pimax value of < 36cmH_2_O (p < 0.001). The OR of presenting with ICU-acquired weakness and not reaching 36cmH_2_O was 9.48 (95%CI = 2.53 - 35.4; p < 0.001). According to the cut-off value chosen (< 36cmH_2_O), a sensitivity value of 31.8% (95%CI = 18.1 - 45.6) was obtained, a specificity value of 96.6% (95%CI = 91 - 100), an LR+ of 7.11 (95%CI = 2.17 - 23.3), and an LR- of 0.71 (95%CI = 0.57 - 0.90) were needed to correctly classify the patients with ICU-acquired weakness as diagnosed using the mss-MRC.

The mean lower limit of normal was 60.3 (± 9.8) cmH_2_O, and the maximum and minimum predicted values were 84.7 and 46.5cmH_2_O, respectively. No predicted Pimax value was below the cut-off point defined in the literature (Pimax < 36cmH_2_O) for any patient.^(19)^

## DISCUSSION

The most relevant finding of the current study was the independent association between delirium and the development of ICU-acquired weakness. Thus far, no evidence has directly linked delirium with weakness.^(25)^ Despite the lack of direct data, increasing evidence has described common factors and outcomes among both conditions. Thus, patients who are delusional or develop ICU-acquired weakness are more likely to have a greater use of sedation, more days of invasive MV, longer stays in the ICU and hospital, and higher mortality rates in the ICU and hospital 1 year after discharge.^(26-30)^ This finding acquires a greater importance considering that the muscle strength assessment was performed only in patients who were alert (i.e., those with RASS from 1 to -1) and aware (i.e., those who fulfilled 4 of 6 commands). As such, we believe that unidentified delirium precluded the possibility of obtaining a low mss-MRC value. Another meeting point exists between both conditions: early ICU mobility as a treatment strategy to avoid the development of ICU-acquired weakness and the onset of delirium to reduce its impact.^(31)^ This meeting point supports the proposed theory in which we suggest that both conditions can be causally associated and should be studied in greater detail together.

As expected, the mean age of patients who had ICU-acquired weakness was significantly higher and was an independent factor that favored the development of this clinical picture. Elderly people can develop sarcopenia, which is further aggravated in those admitted to the ICU^(32)^ and can act as the cause or aggravating factor with regard to the weakness found.^(33)^

Sustained hyperglycemia > 3 days was an independent factor for the development of ICU-acquired weakness. Bercker et al.^(34)^ described similar findings when observing that patients with high daily blood glucose levels developed ICU-acquired weakness. We also know that systematically avoiding hyperglycemia through the implementation of continuous correction therapy with insulin significantly reduces the risk of developing ICU-acquired weakness as well as the days of invasive MV and the length of stay in the ICU.^(35,36)^ The observed relationship between insulin therapy and the lower development of ICU-acquired weakness might justify the association between hyperglycemia and the increased risk for developing ICU-acquired weakness observed among our patients.

This study found a lower mortality rate among patients with or without ICU-acquired weakness than that published by other studies.^(37,38)^ Similarly, the Acute Physiology and Chronic Health Examination (APACHE) score was also lower than those of other similar studies.^(29,39)^ This finding might explain the low mortality rate associated with patients with ICU-acquired weakness. We also believe, as suggested by several authors, that the diagnosis of weakness based on the mss-MRC is applicable to patients who achieve a certain degree of alertness and comprehension, whereas its application is limited in comatose patients or those with sedoanalgesia.^(25,40)^

On the other hand, similar to what other authors have reported, we observed a significant association between patients with inspiratory muscle weakness and ICU-acquired weakness.^(19,37)^ Because assessment via the mss-MRC requires co-workers and conscious patients, an alternative might be the assessment of respiratory muscles because this method can be dispensed at will (see the maneuver described by Marini to evaluate Pimax with a unidirectional valve).^(21)^

The association between limb weakness and respiratory muscle weakness was explored in two previous studies. De Jonghe et al.^(17)^ used the median of their sample and established a value of 30cmH_2_O, which was associated with ICU-acquired weakness. Tzanis et al.^(19)^ defined Pimax as 36cmH_2_O and diagnosed inspiratory weakness in patients with ICU-acquired weakness, with a sensitivity of 88% and a specificity of 76%.

In our patients, the sensitivity was considerably lower, but the specificity values were higher. According to our findings, this difference suggests that a Pimax of ≥ 36cmH_2_O is more useful to exclude respiratory weakness and less useful as a monitoring method for the early diagnosis of ICU-acquired weakness.

In conclusion, the incidence found is similar to that reported so far and varies according to the adopted definition of ICU-acquired weakness, the diagnostic modality, and the characteristics of the included population.^(3,6,41,42)^ The relatively high-incidence density suggests a phenomenon that must be monitored daily. For this purpose, we suggest using simple, non-invasive diagnostic methods and reserving the most invasive methods only for those who cannot have their peripheral muscles assessed using the mss-MRC.

The results found should be validated in the general population to discern possible local biases and the reproducibility of the phenomena found.

The study has limitations. The first is the design; being a single center study, the findings might be due to local biases. For example, poor adherence to protocols might prevent the development of ICU-acquired weakness. The findings must be replicated before generalizing them to the general population. Another clear limitation arises from the tool chosen to diagnosis ICU-acquired weakness (i.e., the mss-MRC), which cannot be using among patients with altered consciousness or those who cannot execute simple instructions. As a result, we believe that the incidence of ICU-acquired weakness might have been underestimated because of this difficulty.

Another limitation was the lack of diagnostic confirmation via diagnostic scaling (muscle biopsy or electromyogram) as suggested by Latronico et al.^(9)^ to discern the type of condition and differentiate muscular involvement from neural involvement or both; these methods can be used to identify the origin of the weakness in more detail.

## CONCLUSION

The intensive care unit acquired weakness is a condition with a high incidence in our environment. Delirium, age, sustained hyperglycemia, and mechanical ventilation > 5 days were independently associated with the development of intensive care unit-acquired weakness. The Pimax of patients with clinical diagnoses of acquired intensive care unit-acquired weakness was significantly reduced, and the limit of 36cmH_2_O showed a high diagnostic value, which excludes the presence of inspiratory weakness associated with intensive care unit-acquired weakness.

## APPENDIX

**Appendix 1:** The muscle strength scale of the Medical Research Council^(18)^

0:	No muscle contraction detected
1:	Fasciculation barely noticeable or traces of contraction
2:	Active motion with gravity removed
3:	Active movement against gravity
4:	Active movement against gravity and some resistance
5:	Active movement against gravity and full resistance
